# Supplementary material for: Construct Validity of the Staff Resource Adequacy Questionnaire for Healthcare Professionals (SRAQ-HP): An Exploratory and Confirmatory Factor Analysis from Latvia
Source: Nurs Rep. 2025 Nov 10;15(11):395. doi: 10.3390/nursrep15110395 (PMC12655560; doi:10.3390/nursrep15110395)
Supplement: Supplementary file 1 [file nursrep-15-00395-s001.zip › nursrep-3921098-supplementary.pdf]

Supplementary Table S1. Staff Resource Adequacy Questionnaire for Healthcare Professionals (SRAQ-HP)

Nr.	Item
	Staffing Adequacy and Workload
1.	There are enough staff in our department during shifts to provide the necessary patient care.
2.	The workload is reasonable and allows me to perform my care tasks to the fullest extent.
3.	Our department has sufficient staff to provide the necessary care for patients.
4.	I don't have to take on the duties of other colleagues due to staff shortages.
5.	I am able to devote sufficient time to the individual needs of each patient.
6.	There are enough staff members to ensure that all tasks are completed fully and on time.
7.	My department has clearly defined staff priorities to help manage the workload, especially during overload periods.
8.	We have enough staff to avoid work overload.
9.	Our department always has enough staff to complete daily tasks.
	Quality of Care
10.	The adequacy of staffing in our department significantly improves the quality of care and patient safety.
11.	The current number of staff ensures that all care tasks are performed adequately.
12.	Patients in our department receive high-quality care, provided there are sufficient staff resources.
13.	I can provide safe care when there are enough staff in the department.
14.	Staff shortages negatively impact patient satisfaction and care outcomes.
15.	I have enough time to provide emotional support to patients.
16.	Work overload does not affect my ability to provide quality care.
	Working Conditions and Support
17.	The hospital provides sufficient support and resources to perform care tasks effectively.
18.	Management responds actively to issues of work overload and provides support when needed.
19.	The hospital offers opportunities to acquire additional skills and knowledge to help manage the workload.
20.	I often receive the necessary support from colleagues, which improves the quality of my work.
21.	The work schedule in our department is designed to avoid staff shortages during shifts.
22.	I feel that the schedule is flexible and takes my personal needs into account.
23.	Management takes measures to ensure the health and safety of employees at work.
24.	Management is often involved in responding to suggestions from employees on how to improve work resources.
25.	Our department has support mechanisms in place, such as the opportunity to consult with colleagues or management, in case psychological support is needed due to staff shortages.
26.	There are incentive programmes and bonuses to help improve staff retention and satisfaction.
